# Patients’ assessment of chronic illness care: a validation study among patients with type 2 diabetes in Finland

**DOI:** 10.1186/s12913-018-3206-7

**Published:** 2018-06-05

**Authors:** Nina Simonsen, Anne M. Koponen, Sakari Suominen

**Affiliations:** 1 Folkhälsan Research Center, P.O. Box 211, 00251 Helsinki, Finland; 2 Department of Public Health, University of Helsinki, P.O. Box 20, 00014 Helsinki, Finland; 3 University of Skövde, Skövde, Sweden; 4 Department of Public Health, University of Turku, Lemminkäisenkatu 1, 20014 Turku, Finland

**Keywords:** Chronic care model, PACIC, Primary care, Quality of care, Type 2 diabetes, Validation

## Abstract

**Background:**

To meet the challenges of the rising prevalence of chronic diseases, such as type 2 diabetes, new approaches to healthcare delivery have been initiated; among these the influential Chronic Care Model (CCM). Valid instruments are needed to evaluate the public health impact of these frameworks in different countries. The Patient Assessment of Chronic Illness Care (PACIC) is a 20-item quality of care measure that, from the perspective of the patient, measures the extent to which care is congruent with the CCM. The aim of this study was to evaluate the psychometric properties of the Finnish translation of the PACIC questionnaire, in terms of validity and reliability, in a large register-based sample of patients with type 2 diabetes.

**Method:**

The PACIC items were translated into Finnish in a standardized forward-backward procedure, followed by a cross-sectional survey among patients with type 2 diabetes (response rate 56%; *n* = 2866). We assessed the Finnish version of the PACIC scale for the following psychometric properties: content validity, internal consistency reliability, convergent and construct validity. We also present descriptive data on total scale as well as predetermined subscale levels.

**Results:**

The item-response on the PACIC scale was high with only small numbers of missing data (0.5–1.1%). Ceiling effects were low (0.3–5.3%) whereas floor effects were over 20% for two of the predetermined subscales (problem solving and follow-up/coordination). The total PACIC scale showed a reasonable distribution and excellent internal consistency (alpha 0.94) while the internal consistency of the subscales were at least acceptable (0.74–0.86). The principal component analysis identified a two- or three-factor solution instead of the proposed five-dimensional. In other respects, the PACIC scale showed the hypothesized relationships with quality of care and outcome measures, thus demonstrating convergent and construct validity.

**Conclusion:**

A Finnish version of the PACIC scale is now validated in the primary care setting among patients with type 2 diabetes. The findings suggest comparable psychometric properties of the Finnish scale as of the original English instrument and earlier translations, and reasonable levels of validity and reliability.

## Background

The rising prevalence of chronic diseases such as type 2 diabetes, worldwide, puts increasing pressure on health systems and especially on primary health care. New models of service delivery focusing on patient-centered and coordinated care have been initiated aiming at improving the quality of care for persons with chronic illnesses, which is a political priority in many countries [1] and endorsed by the WHO [2]. The influential Chronic Care Model (CCM) [3] provides a promising framework to enhance evidence-based chronic care [4]. It describes a patient-centered care approach that is also planned and proactive population-based, and thus different from a reactive acute-oriented care. The evidence concerning the potential of the model, or components of it, to improve care processes, outcomes of care and health care resource use is growing [1, 5] and the model has been proposed as an effective framework in primary care for improving quality of diabetes care [6–8]. The principles of the CCM have been included in disease management programs in different countries, for example, the USA, Canada, England and Australia [1] and, accordingly, in different health-care systems.

In evaluating the public health impact of new frameworks – like the CCM – in health care, adequate instruments, that is, measures of quality that are reliable and valid, are needed [9]. Moreover, instruments covering the patient perspective to quality of care are crucial [10–12]. The Patient Assessment of Chronic Illness Care (PACIC) has been designed to assess quality of care for patients with a chronic illness [13]. It measures the different dimensions of the CCM from the perspective of the patient, focusing on self-management support – including collaborative goal setting, problem solving and follow-up – as well as planned proactive care.

The PACIC scale was developed and validated by Glasgow et al. in the USA for patients with a variety of chronic diseases [13] and for patients with diabetes type 2 [14]. It has been translated and validated into Dutch, Spanish, Danish, French, Spanish [9, 15–17] and German (PACIC-5a) [18]. The psychometric performance of the English scale has been studied also outside USA: in Australia and the UK [12, 19]. In a study comparing different generic instruments, the PACIC was evaluated being among the most promising as regards patients’ experience of quality of integrated care [11].

The Finnish Ministry of Social Affairs and Health proposes implementation of the CCM in primary healthcare centers [20], and as a Finnish validated version of the PACIC scale was not available and earlier studies have suggested the need for validating the scale when adapting it to different healthcare systems, the aim of our study was to evaluate the psychometric properties of the Finnish translation of the PACIC, in a large register-based sample of patients with type 2 diabetes, in terms of reliability and validity.

## Methods

### Design and setting

We performed a standardized translation of the PACIC instrument, followed by a cross-sectional survey among type 2 diabetes patients. This study is part of a larger study of quality of care in diabetes type 2 in five municipalities in Southern and Central Finland (the ‘Good Diabetes Care’ – Study), with a sample from the register of the Social Insurance Institution of Finland (SII). SII is a government agency in charge of settling benefits under national social security programs. SII keeps a countrywide register of all those persons who have entitlement to a special reimbursement for medicines because of chronic diseases, such as diabetes. The sample of the present study was collected among persons who fulfilled the following inclusion criteria:

- had entitlement to a special reimbursement for medicines used in the treatment of type 2 diabetes (ICD-10 code, E11) in 2000–2010, and the right was valid in September 2011 and onward,
- born in 1936–1991 (20–75 years), alive and had no safety prohibition at the time of the data collection,
- Finnish as native language,
- one of the five study municipalities as place of residence.

### Study population

Data collection was done as a postal survey. In all, 7575 persons fulfilled the inclusion criteria and a sample of 5167 persons was collected based on power-analysis: 2000 persons from each of the two large municipalities by random sampling, and all persons from the three small municipalities. There were 2962 (57%) men and 2205 women (43%) in the sample, corresponding to the rate of sex in the total population of patients with type 2 diabetes in the five study municipalities. The questionnaire, including the Finnish version of PACIC together with other quality of care measures as well as demographic and clinical variables, was mailed to respondents in September 2011 by the SII with a reply-paid envelope addressed to the research institute. A reminder to non-respondents was mailed in October, and another reminder with a new copy of the questionnaire in November. The final response rate was 56% (*n* = 2866). The study was approved by the Ethical Committee of the Hjelt Institute, University of Helsinki, and the SII.

### PACIC questionnaire

The PACIC scale [13] (see Table 2) includes 20 items, comprising five subscales: *patient activation* (items 1–3), *delivery system design/decision support* (items 4–6), *goal setting/tailoring* (items 7–11), *problem solving/contextual* (items 12–15) and *coordination/follow-up* (items 16–20). The subscales were not separated in the questionnaire, and, moreover, the 6-month time frame was extended to 12 months – thus patients could base their responses on a longer period of care [21]. Each item is rated on a five point scale (from ‘almost never’ to ‘almost always’). Higher scores indicate higher quality of care. Each subscale is scored by averaging items completed within the scale, and the overall PACIC score is an average across all 20 items.

The English version of the PACIC questionnaire was translated into Finnish in a structured procedure, including forward and backward translations by different translators. The back-translated English version was compared with the original version in English – showing high correspondence – and thereafter a panel of three researchers discussed the translations, which resulted in a slight revision of the original Finnish translation in order to enhance clarity and cultural equivalence.

### Measures administered to assess construct validity

We measured empowerment with the Diabetes Empowerment Scale-Short Form (DES-SF): an 8-item scale that provides an overall assessment of diabetes-related psychosocial self-efficacy [22, 23] on a 5-point scale ranging from ‘strongly disagree’ to ‘strongly agree’, with a Cronbach’s alpha reliability of 0.86 in our data.

We included the Perceived Competence Scale (PCS) measure [24] to assess perceived self-care competence as regards diabetes: a 4-item scale that assesses felt competence for diabetes management. In our study, we used a 5-point scale ranging from ‘strongly disagree’ to ‘strongly agree’, with a Cronbach’s alpha reliability of 0.93 in our data [25].

Self-reported health was measured on a single item 5-point scale, ranging from excellent to poor.

We used the Modified/Short Form Health Care Climate Questionnaire (HCCQ) [24] to assess convergent validity, a subtype of construct validity. The HCCQ assesses the degree to which patients perceive their health professional to be autonomy supportive (versus controlling). The scale has 6 items, and we used a 5-point scale ranging from ‘strongly disagree’ to ‘strongly agree’, with a Cronbach’s alpha reliability of 0.95 in our data [25].

### Analyses

We assessed the Finnish PACIC scale based on quality criteria for questionnaires [15, 26, 27] for the following psychometric properties: content validity, internal consistency reliability, convergent and construct validity. We also present descriptive data on predetermined subscale and total scale levels. The findings are compared with findings from international validation studies.

The content validity of the PACIC is based on the CCM and its aims [13]. We assessed the acceptability and the interpretability of the translated items by exploring rates of missing data on item level, and assessed the proportion of respondents with the lowest (floor effect) and the highest (ceiling effect) possible scores on scale and predetermined subscale levels. Thus, floor and ceiling effects were measured as the percent of patients who reported a minimum (i.e., 1) or maximum (i.e., 5) score on each subscale and on the total PACIC scale. As floor and ceiling effects are present if a substantial proportion of respondents score at either extreme of range, suggesting that a measure is not sensitive to real differences [26], we also used a stricter criterion on the total PACIC scale (< 1.5 or > 4.5). Effects under 20% were defined as optimal [26].

In terms of reliability, we assessed internal consistency at the scale and predetermined subscale levels. Good internal consistency is needed to justify summarizing of items at both subscale and total scale levels [27]. Cronbach’s alphas between 0.70 and 0.80 have been proposed acceptable and scores over 0.80 as excellent [26]; however, alphas should not exceed 0.95 [27]. Inter-correlations between the predetermined subscales were assessed with Spearman’s rho.

Possible differences in PACIC scores among subgroups (related to demographic and clinical characteristics) were explored with analysis of variance, Kruskal-Wallis or Mann-Whitney U tests, as appropriate. Moreover, the strengths of these associations were assessed with Spearman’s rho.

We analyzed the factorial structure of the PACIC scale in the Finnish context with principal component analysis (extraction criterion: Eigenvalue > 1) as many item-variables were not normally distributed. Earlier studies have found strong correlations between subscales and thus the solution was rotated using Oblimin rotation.

Furthermore, we analyzed convergent and construct validity based on the following hypotheses. We expected that PACIC scores, i.e. the receipt of patient-centered, structured chronic illness care, would be correlated moderately (> 0.40) with perceived autonomy supportiveness [12], i.e. scores on the HCCQ, and also positively correlated to outcomes of care, i.e., diabetes empowerment, self-reported health [19, 28] and perceived self-care competence [29]. Moreover, we expected that patients having continuity of care as regards their diabetes care – that is, a regular primary care physician and/or nurse – would have higher PACIC scores compared to those not being cared for by a regular health care professional.

## Results

Responses were received from 2866 respondents (response rate 56%). The mean age of respondents was 63.4 (SD 7.8), 55.9% were male and 40.2% had a higher professional educational level. The mean duration of diabetes type 2 was 8.3 years (SD 6.0). Of the respondents, 2511 (87.6%) responded to all 20 PACIC items, and 93.5% to at least 17, and these 2681 respondents were included in the study sample. In this sample, the mean age was 63.2 (SD 7.7), 55.8% were male, 41% had a higher professional educational level and the mean duration of diabetes was 8.3 years (SD 5.9), thus being quite comparable with the whole sample. Municipal primary healthcare centers were the main provider of diabetes care for 77% of respondents; 18% received their care through occupational healthcare services and 4% through private healthcare centers. The majority (75%) used oral diabetes medication. Demographic and clinical data on the study sample as well as the whole sample, in order to discern possible differences, are provided in Table 1.

**Table 1:** Demographic and clinical data

		Study sample *n* = 2681	Whole sample *n* = 2866
Characteristic		Values are % or mean (SD)	
Gender			
Male		55.8	55.9
Age		63.2 (7.7)	63.4 (7.8)
Age			
27 to 54		13.0	12.7
55 to 64		38.7	37.9
65 to 75		48.3	49.4
Professional education			
Upper secondary education (vocational school) or less		59.0	59.8
Higher education (college, polytechnic, university)		41.0	40.2
Marital status			
Single		9.6	9.8
Married/cohabiting		67.0	66.5
Widowed/divorced		23.4	23.7
Duration of diabetes			
1–3 years		19.7	19.5
4–10 years		53.1	52.9
More than 10 years		27.3	27.6
Medication^a^			
Oral drugs only		74.6	74.7
Oral drugs + insulin/insulin only		24.1	24.1
Other (e.g. GLP-1 analog)		1.3	1.2
Service provider responsible for care of diabetes^b^			
Municipal healthcare center		77.2	77.6
Occupational healthcare service		18.4	18.2
Private healthcare center		4.4	4.3
Perceived autonomy support (HCCQ)	range 1–5	3.5 (1.2)	3.6 (1.2)
Perceived competence	range 1–5	4.2 (0.9)	4.2 (0.9)
Diabetes empowerment	range 1–5	4.0 (0.7)	4.0 (0.7)
Self-reported health			
Poor		50.7	50.7
Good		26.6	26.4
Very good		22.7	22.9
Continuity of care			
Regular physician (yes)		74.3	74.5
Regular nurse (yes)		51.5	51.5

^a^1.1% of all respondents (*n* = 32) used no medication for their diabetes (despite being on the SII register)

^b^1% of all respondents (*n* = 30) reported not having visited a doctor/nurse for their diabetes during the last 2 years, and 1.4% (*n* = 40) had a hospital as their main service provider

The item response on the PACIC scale was high with only small numbers of missing values (0.5–1.1%), also in the whole sample (4–6%; Table 2). Floor effects on the subscales were 5.7–24.9%, over 20% for two of the subscales (*problem solving* and *follow-up/coordination*), whereas ceiling effects were low (0.3–5.3%). On the total PACIC scale, floor and ceiling effects were low (2.8/0.1); when having a stricter lower and upper limit of < 1.5 and > 4.5, the effects were 17.8 and 0.9 (Table 3).

**Table 2:** Missing values on PACIC items^a^

Item	Missing % (Study sample; *n = 2681*)	Missing % (Whole sample; *n = 2866)*
1. Asked for my ideas when we made a treatment plan	0.8	4.7
2. Given choices about treatment to think about	0.8	5.3
3. Asked to talk about any problems with my medicines or their effects	0.3	4.5
4. Given a written list of things I should do to improve my health	0.7	5.0
5. Satisfied that my care was well organized	0.7	4.2
6. Shown how what I did to take care of my illness influenced my condition	0.3	4.0
7. Asked to talk about my goals in caring for my illness	0.2	4.4
8. Helped to set specific goals to improve my eating or exercise	0.5	5.0
9. Given a copy of my treatment plan	0.7	5.4
10. Encouraged to go to a specific group or class to help me cope with my chronic illness	0.2	5.0
11. Asked questions, either directly or on a survey, about my health habits	0.3	4.7
12. Sure that my doctor or nurse thought about my values and my traditions when they recommended treatments to me	1.1	6.0
13. Helped to make a treatment plan that I could carry out in my daily life	0.4	5.3
14. Helped to plan ahead so I could take care of my illness even in hard times	0.7	6.0
15. Asked how my chronic illness affects my life	0.3	5.4
16. Contacted after a visit to see how things were going	0.2	5.1
17. Encouraged to attend programs in the community that could help me	0.3	5.4
18. Referred to a dietician, health educator, or counselor	0.4	5.4
19. Told how my visits with other types of doctors, like an eye doctor or surgeon, helped my treatment	0.2	4.8
20. Asked how my visits with other doctors were going	0.3	5.2

^a^Items shown in the original English version; Glasgow et al. [13]

**Table 3:** Descriptive data on subscales and complete PACIC scale (Study sample; *n = 2681*)

Scale	Missing *%*	Floor/Ceiling^a^ *%*	Mean (SD) (range 1–5)	Median (range 1–5)	IQR^c^
Patient activation (3 items; no missing items allowed)	1.5	17.2/4.7	2.54 (1.21)	2.3	1.7–3.3
Delivery system design/decision support (3 items; no missing items allowed)	1.5	5.7/5.3	3.12 (1.06)	3.3	2.3–4.0
Goal setting/tailoring (5 items; 1 missing item allowed)	0	12.7/0.6	2.25 (0.93)	2.2	1.4–2.8
Problem solving/contextual (4 items; 1 missing item allowed)	0.4	20.2/2.6	2.29 (1.10)	2.0	1.3–3.0
Follow up/coordination (5 items; 1 missing item allowed)	0.1	24.9/0.3	1.79 (0.76)	1.6	1.2–2.2
PACIC total score (20 items; 3 missing items allowed)	0.1	2.8/0.1 (17.8/0.9^b^)	2.32 (0.84)	2.3	1.7–2.9

^a^Floor and ceiling effects = percent of respondents attaining minimum or maximum scores (1/5)

^b^Floor and ceiling effects = percent of respondents attaining PACIC total scores < 1.5/> 4.5

^c^Interquartile range (IQR) = first to third quartile

The mean total PACIC score was 2.32 (SD 0.84) and the median 2.3, with an IQR of 1.7–2.9. The total PACIC scale showed a reasonable distribution and approached normal distribution; however, it was moderately skewed (skewness 0.530, kurtosis − 0.248). The subscale means ranged from 3.12 (1.06) for *delivery system design/decision support* to 1.79 (0.76) for *follow-up/coordination* (Table 3).

Alpha reliabilities were acceptable to excellent, and as follows: total PACIC scale 0.94 (20 items), *patient activation* 0.85 (3 items), *delivery system design/decision support* 0.74 (3 items), *goal setting/tailoring* 0.80 (5 items), *problem solving/contextual* 0.86 (4 items) and *follow-up/coordination* 0.74 (5 items).

The inter-correlation (Spearman’s rho) between the subscales was moderate to high, being highest between the *problem-solving* and *goal-setting* scales (0.78) and *goal-setting* and *decision-support* scales (0.71), whereas the *follow-up* scale was the least correlated with the other scales, and lowest with the *patient-activation* scale (0.51). The *goal-setting* (0.91) and *problem-solving* (0.90) scales correlated the highest with the total PACIC scale and the *follow-up* scale the least (0.76).

The subgroup analysis showed differences in total PACIC scores according to gender, age, marital status, medication, duration of disease and service provider (Table 4). However, the strengths of these associations were modest. As concerns patients’ demographic characteristics, age had the strongest association (Spearman’s rho − 0.12) with the total PACIC score, and among clinical characteristics, the strongest association was found between service provider and PACIC (0.14).

**Table 4:** Results for PACIC by demographic and clinical characteristics (Study sample; *n = 2681*)

Characteristic	PACIC Mean (SD)	*P*-value	Spearman’s rho	*P*-value
Gender				
Men	2.36 (0.84)	0.001	− 0.07	0.000
Women	2.26 (0.84)			
Age				
27–54	2.49 (0.89)	0.000^a^	−0.12	0.000
55–64	2.40 (0.87)			
65–75	2.21 (0.84)			
Professional education				
Upper secondary education or less	2.31 (0.84)	0.90	0.01	0.806
Higher education	2.32 (0.84)			
Marital status				
Single	2.42 (0.87)	0.000	−0.10	0.000
Married/cohabiting	2.34 (0.84)			
Widowed/divorced	2.16 (0.80)			
Duration of diabetes				
≤ 3 years	2.41 (0.85)	0.028	− 0.05	0.011
4–10 years	2.32 (0.85)			
> 10 years	2.27 (0.83)			
Medication				
Oral drugs only	2.29 (0.83)	0.001	0.06	0.002
Oral drugs + insulin/insulin only/other	2.41 (0.86)			
Service provider responsible for care				
Municipal healthcare	2.25 (0.82)	0.000^b^	0.14	0.000
Occupational or private healthcare	2.54 (0.89)			

^a^Kruskal-Wallis test ^b^Mann-Whitney U test

Principal component analysis (PCA) identified a two-factor solution, which explained 53% of the variance. When allowing for a third factor (which almost reached the extraction criterion: Eigenvalue > 1), 58% of the variance was explained (Table 5). In the two-factor solution, Factor 1 is ‘shared decision making and self-care support’ and Factor 2 ‘planned care and social support’, whereas in the three-factor solution, Factor 1 is ‘shared decision making and satisfaction’, Factor 2 ‘coordinated care and social support’, and Factor 3 ‘personal goal-setting and problem-solving’. When performing a PCA separately for patients receiving care in municipal healthcare centers and those receiving care in occupational or private healthcare services (data not shown), an identical three-factor solution as in Table 5 was identified among patients in municipal healthcare centers (only the loading values were different) and nearly an identical two-factor solution among patients in occupational or private healthcare services (only one item, no. 4, loaded differently).

**Table 5:** Factor loadings of the PACIC items using Oblimin rotation^c^ (Study sample; *n = 2681*)

	PCA 1^a^		PCA 2^b^		
Predetermined subscales and items	F1	F2	F1	F2	F3
Patient activation					
1. Asked for my ideas when we made a treatment plan	**0.86**		**0.74**		
2. Given choices about treatment to think about	**0.73**		**0.63**		
3. Asked to talk about any problems with my medicines or their effects	**0.76**		**0.73**		
Delivery system design/Decision support					
4. Given a written list of things I should do to improve my health	0.43				**−0.63**
5. Satisfied that my care was well organized	**0.82**		**0.81**		
6. Shown how what I did to take care of my illness influenced my condition	**0.85**		**0.70**		
Goal setting/Tailoring					
7. Asked to talk about my goals in caring for my illness	**0.74**		**0.50**		−0.44
8. Helped to set specific goals to improve my eating or exercise	**0.57**				**−0.61**
9. Given a copy of my treatment plan		0.45			**−0.70**
10. Encouraged to go to a specific group or class to help me cope with my chronic illness		**0.78**		**0.55**	−0.41
11. Asked questions, either directly or on a survey, about my health habits	**0.57**		0.37		−0.41
Problem solving/Contextual					
12. Sure that my doctor or nurse thought about my values and my traditions when they recommended treatments to me	**0.73**		**0.64**		
13. Helped to make a treatment plan that I could carry out in my daily life	**0.51**	0.39			**−0.64**
14. Helped to plan ahead so I could take care of my illness even in hard times	0.35	**0.55**			**−0.60**
15. Asked how my chronic illness affects my life	0.43	0.44			−0.39
Follow-up/Coordination					
16. Contacted after a visit to see how things were going		**0.66**		**0.62**	
17. Encouraged to attend programs in the community that could help me		**0.83**		**0.68**	
18. Referred to a dietician, health educator, or counselor		**0.59**		**0.62**	
19. Told how my visits with other types of doctors, like an eye doctor or surgeon, helped my treatment	0.39	0.33	0.46	**0.50**	
20. Asked how my visits with other doctors were going		**0.61**		**0.74**	

Loadings ≥0.5 are shown in bold

^a^Extraction criteria: Eigenvalues > 1; variation explained 53%

^b^Extraction criteria: three factors set; variation explained 58%

^c^Items shown in the original English version; Glasgow et al. [13]

As regards convergent and construct validity, PACIC total scores correlated well with perceived autonomy supportiveness (Spearman’s rho 0.58) and significantly also with the outcome variables, and among these, most strongly with the Diabetes empowerment scale (0.24; Table 6). The correlations with the two other outcome variables – perceived competence and self-reported health – were 0.19 respective 0.15. Continuity of care, that is, having a regular physician and/or having a regular nurse, was associated with higher PACIC scores, 2.41/2.05 (yes/no; *p* < 0.001) and 2.47/2.14 (yes/no; *p* < 0.001), respectively, and the strength of the associations were 0.19 and 0.20.

**Table 6:** Associations (Spearman’s rho) between PACIC and health care quality and outcome measures (Study sample; *n = 2681*)

Scale	PACIC
Perceived autonomy support (HCCQ)	0.58***
Continuity of care (no/yes)	
Regular physician	0.19***
Regular nurse	0.20***
Perceived competence	0.19***
Diabetes empowerment	0.24***
Self-reported health (poor/good)	0.15***

***p < .001

## Discussion

Quality improvement in healthcare services, especially in primary health care – in order to answer the challenge of a rising prevalence of chronic conditions within the population – is a focus for health policy makers in many countries. International quality improvement models and measures ensure possibilities to learn from each other, both concerning strengths and weaknesses of quality improvement efforts. To be able to track changes in standards of care, as well as to assess the effectiveness of interventions, good measures are needed [12]. As concerns patients with chronic conditions, their evaluation of care quality and improvements in care quality are important, meaning that measures that assess specifically patients’ perceptions are crucial. In this study, we have assessed the validity and reliability of a Finnish translation of the internationally validated PACIC scale, as well as its utility, in the Finnish healthcare system.

In summary, our findings showed that the translated PACIC scale had a reasonably good validity and reliability among patients with type 2 diabetes in the Finnish primary care setting. The study had a satisfactory response rate and the majority (88%) of respondents answered all PACIC items, indicating good face validity. The validation analyses, moreover, showed that scores on the total scale were reasonably well distributed and the internal consistency was excellent. Two of the five predetermined subscales had problems with floor effects, but all these five subscales had acceptable to excellent internal consistency. In terms of construct validity, the translated PACIC scale, as hypothesized, had significant associations with care quality, i.e., perceived autonomy supportiveness – indicating convergent validity – and continuity of care, as well as outcome measures. The PCA, however, revealed a two- or three-factor structure in the current Finnish healthcare context, instead of the proposed five-dimensional.

In the majority of earlier studies, the five dimension structure of the PACIC scale has not been confirmed. Studies in different populations and healthcare systems have suggested also one-, two- and four-dimensional structures [17, 19, 30–33]. Differences in the PACIC scale structure in different studies have been attributed to methodological differences, but also to real differences between healthcare systems and samples of patients [17]. Spicer and colleagues [21] have raised the issue whether the PACIC scale is a formative rather than a reflective measure, and thus questioned the suitability of factor analysis and internal reliability estimates. Cramm and Nieboer [34], based on their findings in a follow-up study, however, argue that the scale can be regarded a reflective measure. Fan et al. [33] suggest that a universally applicable factorial structure might not exist. In our study, we found different factorial structures among patients receiving care by different healthcare providers. This might suggest differences in care structures and processes, or, alternatively, as suggested by Fan et al. [33], different priorities as concerns chronic disease care among the patients. Some earlier studies have raised questions about the utility of the PACIC subscales, and propose the use of the PACIC total score as an overall experience of chronic illness care [14, 30, 33, 35]. Primary care personnel’s perceptions of implementation of the CCM components seem to be only weakly, though for the most part consistently, associated with patients’ perceptions of CCM (PACIC and its subscales) [36]. More research is needed to determine the degree to which PACIC and possibly the subscales are related to patient outcomes. Moreover, comparing the relative contribution of the predetermined subscales in this regard with the contribution of subscales derived from exploratory factor analysis in the patient population of interest could be worthwhile.

Although the five dimension factorial structure was not established, the predetermined subscales, as well as the total PACIC scale, had good internal consistencies: Cronbach’s alpha being 0.94 for the total scale, and varying from 0.74 to 0.86 for the subscales, thus confirming the results of the original English version [13]. As in our data, the subscales *delivery system design/decision support* and/or *follow-up/coordination* have had the lowest internal consistencies in earlier validation studies as well [12, 13, 15, 18, 31], suggesting that this does not reflect the translation process nor the Finnish primary healthcare context [12].

The mean scores on the total PACIC scale and the subscales were relatively low in our sample and comparable with the scores in patients with type 2 diabetes in Denmark [37] and patients with long-term conditions in UK [12]; in general, lower than those reported elsewhere. Consistent with earlier studies [12, 13], especially *follow-up/coordination* activities were rated low, showing problems with floor effects, as did also the *problem solving* subscale in our study. According to Glasgow and colleagues [13], these two subscales, as well as the *goal setting* scale, form the core of modern chronic care, but are seldom present in the absence of specific quality improvement efforts. Although there have been care quality improvement initiatives in primary healthcare in Finland, there were still ongoing development work to implement, specifically, the Chronic Care Model at the time when the questionnaires in this study were answered, and only in selected healthcare centers. This might explain the low scores and floor effects on the two subscales. Also, when comparing different studies it has to be kept in mind that there are two main versions of the scale. In our study, as in the original study [13], the PACIC scale is rated from ‘almost never’ to ‘almost always’; the other main version applied, extends from ‘never’ to ‘always’. Moreover, as commented earlier [12], the clinical significance of differences in scores is not known.

The subgroup analysis revealed significant associations between PACIC scores and demographic (gender, age, marital status) as well as clinical (duration of disease, medication, service provider) characteristics; only education was not significantly associated. However, these associations were weak (≤ 0.14) and, thus, it is possible that the statistical significance reflects the larger sample size in our study. Nevertheless, earlier findings are inconsistent, also regarding direction of associations. Accordingly, it is unclear whether the scale functions differently in different subgroups and countries or whether there are differences in care quality or expectations. It has to be kept in mind that the findings we report are from unadjusted bivariate analysis, as has mostly been the case also in earlier validation studies.

As regards convergent validity, the PACIC score was – as hypothesized and consistent with earlier studies [12] – associated with perceived autonomy support, an established measure of quality of chronic care [24]. Moreover, the findings showed the hypothesized relationships with continuity of care and outcome measures, thus confirming the construct validity of the PACIC scale, as well as of its Finnish translation. As there has recently been calls for revisions of the PACIC scale because of changes in chronic illness care during the last decade, for example, technological advances [35], we suggest that another way forward might be to complement the PACIC scale with other quality indicators.

Our findings are limited by the cross-sectional nature of the study, meaning that we were not able to assess all aspects of validity and reliability of the PACIC questionnaire. Thus, we did not assess reproducibility (test-retest reliability) or responsiveness. Moreover, we did not interview patients to explore their views on, and understanding of, the translated PACIC scale and its items, though the questionnaire, including the PACIC scale, was tested in a pilot study with possibilities for patients to add comments. Still, the study has a number of strengths, including a large register-based sample of patients with type 2 diabetes, receiving care in different healthcare settings.

## Conclusion

This study contributes to the current evidence of the utility of the PACIC scale in evaluating chronic illness care, and confirms and extends earlier findings regarding convergent and construct validity of the total PACIC scale. The findings suggest comparable psychometrics properties of the Finnish version of the PACIC questionnaire as of the original English instrument and earlier translations, and reasonable levels of validity and reliability among patients with type 2 diabetes in the Finnish primary care setting. Although high floor effects might affect responsiveness, indicating further evaluation of the response categories would be needed, the findings suggest that the translated version of the PACIC scale could be a useful tool for evaluating chronic illness care in Finland.

## Abbreviations

CCM: Chronic Care Model
IQR: Interquartile range
PACIC: Patient Assessment of Chronic Illness Care
PCA: Principal component analysis
SD: Standard Deviation
SII: Social Insurance Institution of Finland
